# Long- and short-distance signaling in the regulation of lateral plant growth

**DOI:** 10.1111/ppl.12103

**Published:** 2013-10-16

**Authors:** Klaus Brackmann, Thomas Greb

**Affiliations:** Gregor Mendel Institute of Molecular Plant Biology, Austrian Academy of SciencesDr. Bohr-Gasse 3, AT-1030, Vienna, Austria

## Abstract

Lateral growth of shoot and root axes by the formation of secondary vascular tissues is an instructive example for the plasticity of plant growth processes. Being purely postembryonic, lateral growth strongly depends on environmental input and is tightly regulated by long- and short-distance signaling. In general, plant vasculature represents the main route for long-distance transport of compounds throughout the plant body, thereby providing also a fast and efficient signaling pipeline for the coordination of growth and development. The vasculature consists of three major tissues; the xylem conducts water and nutrients, the phloem transports mainly organic compounds and the vascular cambium is a group of undifferentiated stem cells responsible for the continuous production of secondary vascular tissues. Notably, the close proximity to functional vascular tissues makes the vascular cambium especially accessible for the regulation by long-distance-derived signaling molecules as well as by the physical and physiological properties of transport streams. Thus, the vascular cambium offers unique opportunities for studying the complex regulation of plant growth processes. In this review, we focus on recent findings about long- and short-distance signaling mechanisms regulating cambium activity and, thereby, lateral expansion of plant growth axes by the formation of additional vascular tissues.

## Introduction

In multicellular organisms, communication among cells is essential for coordinated growth and development. In plants in particular, the flexible regulation of cellular properties by cell-to-cell communication is important throughout the whole life cycle. This is because plants cannot escape from adverse conditions and continuously need to adapt their growth and development to a changing environment. The basis of this growth plasticity is the activity of local stem-cell niches located at the tips and along the flanks of plant growth axes called meristems. Plant meristems provide protective environments that allow maintenance and proliferation of embedded stem cells. Regulation of these meristems is mediated by a combination of receptor–ligand signaling systems (Aichinger et al. 2012). Ligands travel along symplastic or apoplastic routes and bind to receptors sitting in the plasma membrane, in the cytosol or in the endomembrane system. In addition, more direct effectors like transcriptional regulators travel symplastically along plasmodesmata establishing continuity between the cytoplasm of neighboring cells, and non-cell autonomously induce or repress the expression of their target genes.

**Fig 1:**
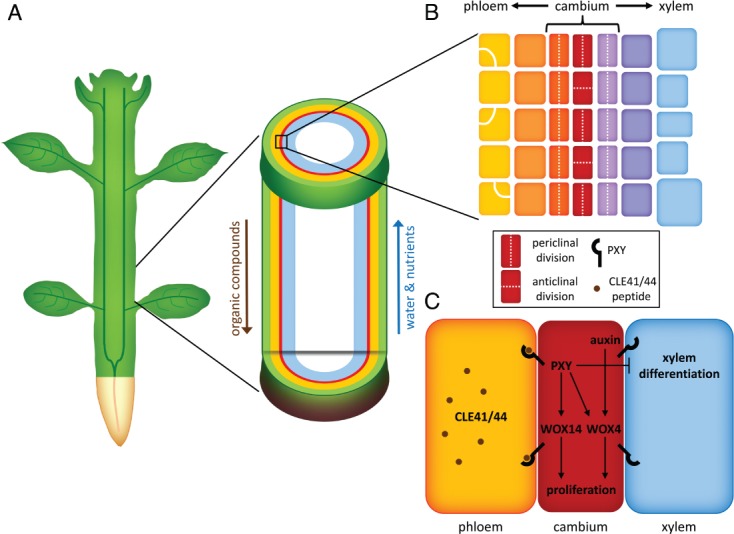
Characteristic anatomy and regulation of the secondary vasculature in dicotyledonous plants. (A) Schematic representation of vascular tissue organization in the mature shoot. (B) Schematic representation of the cambium area at cellular resolution. (C) Short-distance regulation of cambial activity by the CLE41/44-PXY-WOX4/14 signaling module.

The cambium is a meristematic tissue in which the stem cells are a priori arranged in a single-cell layer that forms a closed cylinder along the periphery of stems and roots (Sanchez et al. 2012, Fig. 1A, B). These stem cells, which are also called initials, divide, thereby renewing themselves and providing cells for secondary xylem toward the center of the stem (adaxially) and secondary phloem toward the outside (abaxially, Fig. 1B). Thus, in a first approximation the cambium can be considered as a collection of concentric cylinders of cell layers with different cell identities and degrees of differentiation but which are still dividing. In light of the complex anatomy and growth dynamics, intensive communication between cambium cells harboring different states is essential. However, a fine mapping of cell states and functional subdomains within the cambium area and a detailed description of their mutual interactions are still pending. In particular, the mechanisms balancing the bidirectional recruitment of cells into new layers of secondary vasculature have hardly been touched so far. This lack of knowledge is remarkable considering the essential role of lateral growth for plant performance and terrestrial biomass accumulation.

## Communication between growing organs – the long-distance interaction between the shoot apex and the cambium

Auxin has been extensively characterized in the context of long-distance regulation of lateral growth. The key observation is that decapitation of shoots results in the loss of cambial activity, which however can be restored by the apical application of auxin (e.g. Ko et al. 2004). Further support for an important role of auxin in lateral growth came from direct auxin measurements in *Populus* and *Pinus* trees. In both species, the concentration of the major endogenous auxin, indole-3-acetic acid (IAA), peaks in the center of the cambial zone and declines to both sides toward the xylem and phloem (Uggla et al. 1996, 1998). This observation led to the idea that auxin determines cell fate during lateral growth in a dose-dependent manner (Bhalerao and Bennett 2003). Collectively, it is believed that auxin is mainly biosynthesized in the shoot apex, transported basipetally along the shoot via the cambium and/or the phloem (Lachaud and Bonnemain 1984) and distributed laterally via polar auxin transport across the cambial zone. Consistent with this model, various genes encoding for auxin transporters like PIN-FORMED (PIN) efflux and AUXIN RESISTANT 1 (AUX1)-like influx carriers are differentially expressed across the cambial zone in *Populus* (Schrader et al. 2003). However, more direct evidence for lateral transport of auxin within the cambium area is missing.

Besides polar auxin transport, auxin signaling provides another regulatory layer in cambium regulation. Similar to auxin transporters, transcription of genes encoding for auxin signaling components showed a strong positive correlation with the pattern of auxin levels across the cambium area in *Populus* (Schrader et al. 2003). However, the expression maxima of auxin-responsive genes were rather found in the developing xylem (Nilsson et al. 2008). This indicates not only a role for auxin in xylogenesis but also an indirect relationship between auxin concentration and transcriptional activation of most of the auxin-responsive genes that are expressed in the cambium area. Consistent with this observation, xylem cell expansion was more sensitive than cambium proliferation to reduced auxin responsiveness, which was achieved by expressing a transcriptional repressor of auxin signaling (*PttIAA3*) throughout the plant (Nilsson et al. 2008). Interestingly, trees overexpressing *PttIAA3* also showed an enlarged zone of anticlinal divisions (Nilsson et al. 2008) usually characteristic of cambial initial cells (Fig. 1B, Schrader et al. 2004). This observation may indicate that auxin signaling not only positively regulates cambium activity but also spatially restricts stem-cell characteristics in the cambium area.

How is auxin signaling translated into cambium activity? Lately, it was demonstrated that strigolactone (SL) signaling directly in the cambium area is important in this respect (Agustí et al. 2011a). This conclusion was based on the observation that cambium sensitivity to auxin was reduced in SL biosynthesis and signaling mutants. Conversely, treatments with the SL analog GR24 resulted in the activation of cambium activity at a similar rate in wild-type and *auxin-resistant1* (*axr1*) mutants impaired in auxin signaling, suggesting that auxin signaling is not required for an SL effect on cambium activity (Agustí et al. 2011a). Furthermore, restoring SL signaling specifically in the cambium of the SL signaling mutant *more axillary branches2* (*max2*) was sufficient for complementing defects in cambium activity. These findings argue for a role of local SL signaling downstream of the auxin signaling pathway in lateral growth regulation.

## Gibberellins and brassinosteroids: candidates for balancing the formation of cambium-derived tissues

Gibberellins (GAs) are important endogenous regulators of cambial activity and, in this context, derive presumably from leaves (Dayan et al. 2012). In general, exogenous GA treatments stimulate cell divisions in the cambial region. However, GA-induced cells did not display the typical appearance of cambium cells and also did not differentiate properly in *Populus* (Björklund et al. 2007). This indicates that GA alone is not sufficient for promoting all aspects of lateral growth. Consistently, co-application of auxin and GA led to proper induction and differentiation of cambium cells and a stronger enhancement of cambial activity than either individual hormone treatment, indicating that these two hormones function synergistically (Björklund et al. 2007). This synergy might be explained at least partially by the stimulatory role of GA on auxin transport on one side and the promoting effect of auxin on GA biosynthesis on the other side (Björklund et al. 2007). Analysis of the distribution of bioactive GAs in *Populus* stems showed that, in contrast to auxin, GA levels do not peak in the cambium but in the adjacent domain of xylem cell expansion, supporting a role of GA in the promotion of cell division and differentiation in cells committed to the xylem cell fate (Israelsson et al. 2005). Such a role was further suggested by transgenic trees ectopically expressing GA 20-oxidase, which resulted in enhanced GA levels as well as increased xylem fiber cell number and length (Eriksson et al. 2000). More recently, it was found that GAs also act as a mobile signal in *Arabidopsis* that, upon flowering, is transported from shoots to hypocotyls where it triggers the formation of xylem cells (Ragni et al. 2011).

Brassinosteroids also play a role in the formation of cambium-derived tissues. Exogenously applied brassinosteroids promoted the differentiation of tracheary elements and regulate the expression of xylem-related genes in a xylogenic culture system of *Zinnia* mesophyll cells (Yamamoto et al. 2001 and references therein). Furthermore, gas chromatography–mass spectrometry analysis of the cambial zone of *Pinus* trees identified two bioactive brassinosteroids specifically present in this meristem (Kim et al. 1990). A potential role for brassinosteroids in balancing cambium-dependent tissue production is further supported by an enlarged secondary phloem and reduced secondary xylem in vascular bundles of *Arabidopsis* mutants defective in brassinosteroid biosynthesis and signaling (Caño-Delgado et al. 2004).

## Cytokinins stimulate cambium activity

A role of cytokinins in stimulating cambium activity is well established. In particular, recent studies show that reductions in cambial cytokinin levels, achieved through the expression of cytokinin degrading enzymes or by combining mutations in the cytokinin biosynthesis pathway, substantially impair lateral growth in *Populus* and *Arabidopsis*, respectively (Matsumoto-Kitano et al. 2008, Nieminen et al. 2008). In addition, single and double *Arabidopsis* mutants of the cytokinin receptors ARABIDOPSIS HISTIDINE KINASE2 (AHK2) and AHK3 display reduced (pro)cambial cell numbers and lack secondary growth (Hejatko et al. 2009). The site of cytokinin production does not seem to be crucial in this context because grafting experiments showed that wild-type shoots or roots could restore cambium activity in roots or shoots, respectively, impaired in cytokinin biosynthesis (Matsumoto-Kitano et al. 2008).

The mechanisms mediating endogenous cytokinin function in cambium regulation and their interaction with other signaling pathways are still poorly understood. Transcriptional profiling across the vasculature of *Populus* showed that genes encoding cytokinin receptors and a cytokinin primary response gene (*PtRR7*) are especially active in the cambial zone (Nieminen et al. 2008). Moreover, the His kinase CYTOKININ-INDEPENDENT1 (CKI1), implicated in the perception of cytokinins, is expressed in (pro)cambial cells of the vascular bundles in *Arabidopsis* stems (Hejatko et al. 2009). Reduced *CKI1* transcript levels resulted in reduced (pro)cambial cell divisions similar to *ahk2* single and *ahk2;ahk3* double mutants, indicating that cytokinin signaling in the cambium itself is required for proper cambium function (Hejatko et al. 2009).

## Stress-induced cambium activity: ethylene and jasmonic acid

Ethylene is a gaseous signaling molecule that is important for plant development. Its function in lateral growth regulation has been initially addressed by exogenous hormone application experiments. Both treatment with the ethylene precursor 1-aminocyclopropane-1-carboxylic acid (ACC) and local application of gaseous ethylene to *Populus* stems stimulated lateral growth (Junghans et al. 2004, Love et al. 2009). In addition, it was recently shown that *ethylene overproducer1* (*eto1*) mutants show an increased number of (pro)cambial cells in *Arabidopsis* (Etchells et al. 2012). On the contrary, mutations in the positive regulators of ethylene signaling ETHYLENE INSENSITIVE2 (EIN2), EIN5 and ETHYLENE RECEPTOR1 (ETR1) dramatically enhanced the defect in (pro)cambium proliferation observed in *phloem intercalated with xylem* (*pxy*) mutants (Etchells et al. 2012, see below). Interestingly *ein2*, *ein5* and *etr1* single mutants do not display any alterations in cambial cell number, indicating compensatory functions of ethylene signaling in the absence of PXY (Etchells et al. 2012). The mechanism triggering an ethylene-dependent cambium response is not clear, but the biosynthesis of ethylene in general is activated in response to many environmental cues and particularly increases during the formation of tension wood in response to mechanical stress induced by bending tree stems (Andersson-Gunneras et al. 2003). To address the role of ethylene in tension wood formation, Love et al. (2009) engineered ethylene-overproducing and -insensitive *Populus* lines. These lines displayed increased xylem formation and reduced tension wood formation, respectively (Love et al. 2009). Notably, the ethylene-insensitive trees were not reported to show reduced lateral growth in the absence of bending-induced mechanical stress (Love et al. 2009), arguing that ethylene signaling is not essential for cambium activity under normal growth conditions, but rather becomes active as a response to different stresses.

More recently, it was demonstrated that the jasmonic acid (JA) signaling pathway is involved in the stimulation of cambial activity (Sehr et al. 2010). Initially, it was found that the touch- and JA-inducible repressor of JA signaling *JASMONATE ZIM-DOMAIN10* (*JAZ10*) is active in laterally growing *Arabidopsis* stems (Sehr et al. 2010). Mutants defective in *JAZ10* displayed enhanced cambial activity, whereas mutants impaired in *CORONATINE INSENSITIVE1* (*COI1*) or *JASMONATE INSENSITIVE1* (*JIN1/MYC2*), two positive regulators of JA signaling, showed reduced cambium proliferation (Sehr et al. 2010). Interestingly, Zhu et al. (2011) demonstrated that JA is able to enhance the activity of transcription factors that mediate plant response to ethylene signaling (Zhu et al. 2011). This observation provides a potential framework how mechanosensing through the JA signaling pathway might be converted into enhanced cambial activity via ethylene signaling during reaction wood formation.

## Stem-cell maintenance in the cambium

Despite the substantial anatomical differences between shoot and root apical meristems (SAM and RAM) and the vascular cambium, central molecular mechanisms controlling both meristem types appear to be similar. The first indication in this direction was provided by the discovery of the CLAVATA3/ESR-RELATED (CLE) peptide TDIF (tracheary element differentiation inhibitory factor), which was identified as a repressor of xylem differentiation and promoter of cell proliferation in the *Zinnia* cell culture system (Ito et al. 2006). The two *Arabidopsis* TDIF homologs, CLE41 and CLE44, are expressed in the phloem and secreted into the apoplastic space. In the neighboring (pro)cambium they induce stem-cell proliferation and inhibit tracheary element differentiation (Fig. 1C; Hirakawa et al. 2008, Whitford et al. 2008, Etchells and Turner 2010). This indicates that CLE41/44 signaling mediates both the proliferation of vascular stem cells and the inhibition of xylem differentiation, reminiscent of the situation in apical meristems where other CLE peptides fulfill similar roles (Schoof et al. 2000, Stahl et al. 2009).

CLE41/44 signaling in the vascular stem cells is perceived by the leucine-rich repeat receptor-like kinase (LRR-RLK) PXY, also known as TDR (Fisher and Turner 2007, Hirakawa et al. 2008, Etchells and Turner 2010). PXY is expressed in the *Arabidopsis* cambium and presumably targeted to the plasma membrane. *pxy* mutants display interspersed phloem and xylem tissues and defects in the orientation and number of (pro)cambial cell divisions (Fig. 1C; Fisher and Turner 2007, Hirakawa et al. 2008, Etchells and Turner 2010). Thus, the PXY/CLE41/44 module seems to provide positional information similar to LRR-RLK/CLE-dependent cell-to-cell communication found in SAM and RAM (Schoof et al. 2000, Stahl et al. 2009). However, while in apical meristems the LRR-RLK/CLE signaling modules inhibit meristematic activity, the PXY/CLE41/44 module stimulates cambium proliferation (Hirakawa et al. 2008, 2010), indicating either substantial differences in the regulation of apical meristems and the cambium or the existence of yet uncharacterized signaling components. Support for the latter hypothesis is provided by the identification of additional cambium-specific LRR-RLKs (Agustí et al. 2011b, Bryan et al. 2012, Wang et al. 2013). One of them, MORE LATERAL GROWTH1 (MOL1), indeed acts as a negative regulator of cambium activity (Agustí et al. 2011b).

CLE41/44 signaling stimulates cambium activity by promoting the expression of the *WUSCHEL-RELATED HOMEOBOX4* (*WOX4*) gene in a *PXY*-dependent manner (Hirakawa et al. 2010). *WOX4* activity is essential for maintaining stem-cell fate in the cambium (Hirakawa et al. 2010, Suer et al. 2011) equivalent to the roles of *WUSCHEL* (*WUS*) and *WOX5*, two other members of the *WOX* gene family active in SAM and RAM, respectively (Schoof et al. 2000, Sarkar et al. 2007). Strikingly, *WOX4* expression in the stem is also induced by auxin independently from *PXY* (Suer et al. 2011). Thus, *WOX4* seems to integrate the inputs of long-distance-derived signals acting in parallel to the PXY/CLE41/44 module. Recently, another member of the *WOX* gene family, *WOX14*, was shown to be expressed in the cambium (Etchells et al. 2013). *wox14* single mutants show no alterations in vascular stem-cell number, whereas *wox4;wox14* double mutants display a stronger reduction of vascular cell division than *wox4* single mutants. Therefore, *WOX14* acts, at least partially, redundantly to *WOX4* in mediating (pro)cambium activity (Fig. 1C; Etchells et al. 2013).

The regulatory networks acting downstream of WOX4/14 are still largely uncharacterized. Recent data indicate that the ethylene-responsive ETHYLENE RESPONSE FACTOR (ERF) transcription factors ERF1, ERF018 and ERF109 promote vascular cell divisions downstream of *PXY* and *WOX4*. In *pxy* and *wox4* mutants, activity of the *ERF1*, *ERF018* and *ERF109* genes is enhanced partially masking defects caused by disturbed *PXY* or *WOX4* functions (Etchells et al. 2012). In conclusion, *WOX4* seems to mediate the interaction between auxin and ethylene signaling and integrate also other pathways to control the rate of cell divisions in plant vascular tissue.

## Setting the polarity of the cambium

Whether the strict bidirectional polarity of tissue production by the cambium is the output of a constant signaling process along the radial axis of the cambium zone or whether this is implemented during cambium establishment and then maintained is unclear. However, there are indications that an early establishment of the adaxial–abaxial polarity is crucial. For example, tissue production in tissue blocks from interfascicular regions in *Ricinus communis* maintained its original polarity even when these blocks were excised before any sign of cambium formation and inserted in reversed orientation (Siebers 1971). Importantly, cambium formation happens when surrounding organs have already established an overall adaxial–abaxial polarity (reviewed in Husbands et al. 2009) and factors active in adaxial or abaxial domains presumably transfer spatial information to (pro)cambium cells. For example, class III HOMEODOMAIN-LEUCINE ZIPPER (HD-ZIPIII) transcription factors are important for organ polarization and are expressed in adaxial tissues of young leaves, roots and stems including the developing xylem (e.g. Emery et al. 2003). Reduction of *HD-ZIPIII* expression led to decreased organ polarity and, at the same time, to defects in xylem differentiation (Ilegems et al. 2010). Conversely, KANADI (KAN) transcription factors are expressed in abaxial organ domains including the phloem (Emery et al. 2003). No direct effect of KAN transcription factors on phloem specification and/or differentiation has been reported, but *Arabidopsis* mutants impaired in the activity of all four *KAN* genes display an expansion of the (pro)cambium domain in the center of vascular bundles and increased cambium activity (Ilegems et al. 2010). Enhanced cambium activity has also been reported for *hd-zipIII* mutants (Talbert et al. 1995) and, collectively, these observations are in line with the idea that the antagonism between adaxial and abaxial factors determines cambium identity at the boundary of both domains (Ilegems et al. 2010).

In addition to an inherent polarity of the cambium, it is also possible that phloem and xylem specification is induced or supported by signals derived from differentiated tissues and transported along the growth axis to ensure continuity of vascular transport routes. One candidate for this is xylogen, a proteoglycan-like factor polarly localized in cell walls of differentiating tracheary elements and essential for xylem continuity (Motose et al. 2004). On the other side, OCTOPUS (OPS), a protein of unknown function, is polarly localized to the plasma membrane of provascular and protophloem cells and promotes phloem continuity (Truernit et al. 2012).

## Conclusion

Our current understanding of lateral plant growth regulation is more than fragmentary. This becomes especially obvious when considering the lack of knowledge of how different signaling pathways interact with each other on the level of cambium cells, which signaling components act in different cambium subdomains, how these subdomains interact or how environmental input is integrated. These aspects are particularly difficult to address because lateral plant growth is a late process during plant development and many essential regulators may also have functions during earlier growth phases. Therefore, distinguishing between primary and secondary effects is often challenging when applying standard genetic tools. Moreover, interpreting short-term effects of pharmacological treatments or induced changes of gene activities has to be done with caution as no live cell imaging during lateral growth is possible to date hampering the elucidation of the dynamics of induced changes. Encouragingly, *Arabidopsis thaliana* is becoming established more and more as a model for lateral plant growth and exploitation of genetic and molecular tools available in this reference plant has already provided important novel insights (Ito et al. 2006, Fisher and Turner 2007, Hirakawa et al. 2008, Melzer et al. 2008, Etchells and Turner 2010, Agustí et al. 2011b, Ragni et al. 2011, Suer et al. 2011). These advancements are promising and considering the conservation of key regulators of lateral growth among dicotyledonous species, transferability of findings to a broader spectrum of species is very likely. After all, the integration of individual interactions into comprehensive regulatory models that are also able to reproduce interspecies and intraspecies variations will be essential for establishing a systemic view on a process of such fundamental importance for plant growth and development.

## Abbreviations

ACC: 1-aminocyclopropane-1-carboxylic acid
CLE: CLAVATA3/ESR-RELATED
ERF: ETHYLENE RESPONSE FACTOR
GA: gibberellin
HD-ZIP: HOMEODOMAIN-LEUCINE ZIPPER
IAA: indole-3-acetic acid
JA: jasmonic acid
JAZ10: JASMONATE ZIM-DOMAIN10
KAN: KANADI
LRR-RLK: leucine-rich repeat receptor-like kinase
PXY: PHLOEM INTERCALATED WITH XYLEM
RAM: root apical meristem
SAM: shoot apical meristem
SL: strigolactone
TDIF: tracheary element differentiation inhibitory factor
TDR: TDIF RECEPTOR
WOX: WUSCHEL-RELATED HOMEOBOX

## References

[b1] Agustí J, Herold S, Schwarz M, Sanchez P, Ljung K, Dun EA, Brewer PB, Beveridge CA, Sieberer T, Sehr EM, Greb T (2011a). Strigolactone signaling is required for auxin-dependent stimulation of secondary growth in plants. Proc Natl Acad Sci USA.

[b2] Agustí J, Lichtenberger R, Schwarz M, Nehlin L, Greb T (2011b). Characterization of transcriptome remodeling during cambium formation identifies MOL1 and RUL1 as opposing regulators of secondary growth. PLoS Genet.

[b3] Aichinger E, Kornet N, Friedrich T, Laux T (2012). Plant stem cell niches. Annu Rev Plant Biol.

[b4] Andersson-Gunneras S, Hellgren JM, Björklund S, Regan S, Moritz T, Sundberg B (2003). Asymmetric expression of a poplar ACC oxidase controls ethylene production during gravitational induction of tension wood. Plant J.

[b5] Bhalerao RP, Bennett MJ (2003). The case for morphogens in plants. Nat Cell Biol.

[b6] Björklund S, Antti H, Uddestrand I, Moritz T, Sundberg B (2007). Cross-talk between gibberellin and auxin in development of *Populus* wood: gibberellin stimulates polar auxin transport and has a common transcriptome with auxin. Plant J.

[b7] Bryan AC, Obaidi A, Wierzba M, Tax FE (2012). XYLEM INTERMIXED WITH PHLOEM1: a leucine-rich repeat receptor-like kinase required for stem growth and vascular development in *Arabidopsis thaliana*. Planta.

[b8] Caño-Delgado A, Yin Y, Yu C, Vafeados D, Mora-Garcia S, Cheng JC, Nam KH, Li J, Chory J (2004). BRL1 and BRL3 are novel brassinosteroid receptors that function in vascular differentiation in Arabidopsis. Development.

[b9] Dayan J, Voronin N, Gong F, Sun TP, Hedden P, Fromm H, Aloni R (2012). Leaf-induced gibberellin signaling is essential for internode elongation, cambial activity, and fiber differentiation in tobacco stems. Plant Cell.

[b10] Emery JF, Floyd SK, Alvarez J, Eshed Y, Hawker NP, Izhaki A, Baum SF, Bowman JL (2003). Radial patterning of Arabidopsis shoots by class III HD-ZIP and KANADI genes. Curr Biol.

[b11] Eriksson ME, Israelsson M, Olsson O, Moritz T (2000). Increased gibberellin biosynthesis in transgenic trees promotes growth, biomass production and xylem fiber length. Nat Biotechnol.

[b12] Etchells JP, Turner SR (2010). The PXY-CLE41 receptor ligand pair defines a multifunctional pathway that controls the rate and orientation of vascular cell division. Development.

[b13] Etchells JP, Provost CM, Turner SR (2012). Plant vascular cell division is maintained by an interaction between PXY and ethylene signalling. PLoS Genet.

[b14] Etchells JP, Provost CM, Mishra L, Turner SR (2013). WOX4 and WOX14 act downstream of the PXY receptor kinase to regulate plant vascular proliferation independently of any role in vascular organisation. Development.

[b15] Fisher K, Turner S (2007). PXY: a receptor-like kinase essential for maintaining polarity during plant vascular-tissue development. Curr Biol.

[b16] Hejatko J, Ryu H, Kim GT, Dobesova R, Choi S, Choi SM, Soucek P, Horak J, Pekarova B, Palme K, Brzobohaty B, Hwang I (2009). The histidine kinases CYTOKININ-INDEPENDENT1 and ARABIDOPSIS HISTIDINE KINASE2 and 3 regulate vascular tissue development in Arabidopsis shoots. Plant Cell.

[b17] Hirakawa Y, Shinohara H, Kondo Y, Inoue A, Nakanomyo I, Ogawa M, Sawa S, Ohashi-Ito K, Matsubayashi Y, Fukuda H (2008). Non-cell-autonomous control of vascular stem cell fate by a CLE peptide/receptor system. Proc Natl Acad Sci USA.

[b18] Hirakawa Y, Kondo Y, Fukuda H (2010). TDIF peptide signaling regulates vascular stem cell proliferation via the WOX4 homeobox gene in Arabidopsis. Plant Cell.

[b19] Husbands AY, Chitwood DH, Plavskin Y, Timmermans MC (2009). Signals and prepatterns: new insights into organ polarity in plants. Genes Dev.

[b20] Ilegems M, Douet V, Meylan-Bettex M, Uyttewaal M, Brand L, Bowman JL, Stieger PA (2010). Interplay of auxin, KANADI and Class III HD-ZIP transcription factors in vascular tissue formation. Development.

[b21] Israelsson M, Sundberg B, Moritz T (2005). Tissue-specific localization of gibberellins and expression of gibberellin-biosynthetic and signaling genes in wood-forming tissues in aspen. Plant J.

[b22] Ito Y, Nakanomyo I, Motose H, Iwamoto K, Sawa S, Dohmae N, Fukuda H (2006). Dodeca-CLE peptides as suppressors of plant stem cell differentiation. Science.

[b23] Junghans U, Langenfeld-Heyser R, Polle A, Teichmann T (2004). Effect of auxin transport inhibitors and ethylene on the wood anatomy of poplar. Plant Biol (Stuttg).

[b24] Kim SK, Abe H, Little CH, Pharis RP (1990). Identification of two brassinosteroids from the cambial region of Scots Pine (*Pinus silvestris*) by gas chromatography-mass spectrometry, after detection using a dwarf rice lamina inclination bioassay. Plant Physiol.

[b25] Ko JH, Han KH, Park S, Yang J (2004). Plant body weight-induced secondary growth in Arabidopsis and its transcription phenotype revealed by whole-transcriptome profiling. Plant Physiol.

[b26] Lachaud S, Bonnemain JL (1984). Seasonal variations in the polar-transport pathways and retention sites of [3H]indole-3-acetic acid in young branches of *Fagus sylvatica* L. Planta.

[b27] Love J, Björklund S, Vahala J, Hertzberg M, Kangasjärvi J, Sundberg B (2009). Ethylene is an endogenous stimulator of cell division in the cambial meristem of *Populus*. Proc Natl Acad Sci USA.

[b28] Matsumoto-Kitano M, Kusumoto T, Tarkowski P, Kinoshita-Tsujimura K, Vaclavikova K, Miyawaki K, Kakimoto T (2008). Cytokinins are central regulators of cambial activity. Proc Natl Acad Sci USA.

[b29] Melzer S, Lens F, Gennen J, Vanneste S, Rohde A, Beeckman T (2008). Flowering-time genes modulate meristem determinacy and growth form in *Arabidopsis thaliana*. Nat Genet.

[b30] Motose H, Sugiyama M, Fukuda H (2004). A proteoglycan mediates inductive interaction during plant vascular development. Nature.

[b31] Nieminen K, Immanen J, Laxell M, Kauppinen L, Tarkowski P, Dolezal K, Tahtiharju S, Elo A, Decourteix M, Ljung K, Bhalerao R, Keinonen K, Albert VA, Helariutta Y (2008). Cytokinin signaling regulates cambial development in poplar. Proc Natl Acad Sci USA.

[b32] Nilsson J, Karlberg A, Antti H, Lopez-Vernaza M, Mellerowicz E, Perrot-Rechenmann C, Sandberg G, Bhalerao RP (2008). Dissecting the molecular basis of the regulation of wood formation by auxin in hybrid aspen. Plant Cell.

[b33] Ragni L, Nieminen K, Pacheco-Villalobos D, Sibout R, Schwechheimer C, Hardtke CS (2011). Mobile gibberellin directly stimulates Arabidopsis hypocotyl xylem expansion. Plant Cell.

[b34] Sanchez P, Nehlin L, Greb T (2012). From thin to thick – major transitions during stem development. Trends Plant Sci.

[b35] Sarkar AK, Luijten M, Miyashima S, Lenhard M, Hashimoto T, Nakajima K, Scheres B, Heidstra R, Laux T (2007). Conserved factors regulate signalling in *Arabidopsis thaliana* shoot and root stem cell organizers. Nature.

[b36] Schoof H, Lenhard M, Haecker A, Mayer KF, Jurgens G, Laux T (2000). The stem cell population of Arabidopsis shoot meristems in maintained by a regulatory loop between the CLAVATA and WUSCHEL genes. Cell.

[b37] Schrader J, Baba K, May ST, Palme K, Bennett M, Bhalerao RP, Sandberg G (2003). Polar auxin transport in the wood-forming tissues of hybrid aspen is under simultaneous control of developmental and environmental signals. Proc Natl Acad Sci USA.

[b38] Schrader J, Nilsson J, Mellerowicz E, Berglund A, Nilsson P, Hertzberg M, Sandberg G (2004). A high-resolution transcript profile across the wood-forming meristem of poplar identifies potential regulators of cambial stem cell identity. Plant Cell.

[b39] Sehr EM, Agustí J, Lehner R, Farmer EE, Schwarz M, Greb T (2010). Analysis of secondary growth in the Arabidopsis shoot reveals a positive role of jasmonate signalling in cambium formation. Plant J.

[b40] Siebers AM (1971). Initiation of radial polarity in the interfascicular cambium of *Ricinus communis* L. Acta Bot Neerl.

[b41] Stahl Y, Wink RH, Ingram GC, Simon R (2009). A signaling module controlling the stem cell niche in Arabidopsis root meristems. Curr Biol.

[b42] Suer S, Agustí J, Sanchez P, Schwarz M, Greb T (2011). WOX4 imparts auxin responsiveness to cambium cells in Arabidopsis. Plant Cell.

[b43] Talbert PB, Adler HT, Parks DW, Comai L (1995). The REVOLUTA gene is necessary for apical meristem development and for limiting cell divisions in the leaves and stems of *Arabidopsis thaliana*. Development.

[b44] Truernit E, Bauby H, Belcram K, Barthelemy J, Palauqui JC (2012). OCTOPUS: a polarly localised membrane-associated protein, regulates phloem differentiation entry in *Arabidopsis thaliana*. Development.

[b45] Uggla C, Moritz T, Sandberg G, Sundberg B (1996). Auxin as a positional signal in pattern formation in plants. Proc Natl Acad Sci USA.

[b46] Uggla C, Mellerowicz EJ, Sundberg B (1998). Indole-3-acetic acid controls cambial growth in Scots pine by positional signaling. Plant Physiol.

[b47] Wang J, Kucukoglu M, Zhang L, Chen P, Decker D, Nilsson O, Jones B, Sandberg G, Zheng B (2013). The Arabidopsis LRR-RLK, PXC1, is a regulator of secondary wall formation correlated with the TDIF-PXY/TDR-WOX4 signaling pathway. BMC Plant Biol.

[b48] Whitford R, Fernandez A, De Groodt R, Ortega E, Hilson P (2008). Plant CLE peptides from two distinct functional classes synergistically induce division of vascular cells. Proc Natl Acad Sci USA.

[b49] Yamamoto R, Fujioka S, Demura T, Takatsuto S, Yoshida S, Fukuda H (2001). Brassinosteroid levels increase drastically prior to morphogenesis of tracheary elements. Plant Physiol.

[b50] Zhu Z, An F, Feng Y, Li P, Xue L, A M, Jiang Z, Kim JM, To TK, Li W, Zhang X, Yu Q, Dong Z, Chen WQ, Seki M, Zhou JM, Guo H (2011). Derepression of ethylene-stabilized transcription factors (EIN3/EIL1) mediates jasmonate and ethylene signaling synergy in Arabidopsis. Proc Natl Acad Sci USA.

